# The Pen and Plow

**Published:** 1877-02

**Authors:** 


*The Pen and Plow.*—A monthly Journal devoted to interesting and instructive literature and useful agricultural knowledge. Familiarity with its pages for several years, enables us to recommend it as a desirable publication. Published by Pen & Plow Publishing Co., and Edited by J. Payne Low. Terms $1.00 a year: Ten cents a single copy.

